# Cutaneous pilomatrical carcinosarcoma: a case report with molecular analysis and literature review

**DOI:** 10.1186/s13000-020-0925-y

**Published:** 2020-01-31

**Authors:** Thi My Hanh Luong, Yuko Akazawa, Zhanna Mussazhanova, Katsuya Matsuda, Nozomi Ueki, Shiro Miura, Toshihide Hara, Hiroko Yokoyama, Masahiro Nakashima

**Affiliations:** 1 Department of Tumor and Diagnostic Pathology, Atomic Bomb Disease Institute, Nagasaki University Graduate School of Biomedical Sciences, 1-12-4 Sakamoto, Nagasaki City, Nagasaki 852-8523 Japan; 2 Department of Pathology, National Hospital Organization Nagasaki Medical Center, Omura, 856-8562 Nagasaki Japan; 3 Department of Dermatology, Isahaya General Hospital, Isahaya, Japan

**Keywords:** Carcinosarcoma, Skin, β-Catenin

## Abstract

**Background:**

Cutaneous pilomatrical carcinosarcoma (CS) is a very rare biphasic tumor composed of admixed epithelial and mesenchymal malignant cells, with limited information on its pathogenesis. We report a case of pilomatrical CS of the scalp with comparative immunohistochemical and molecular analysis together with a review of the literature.

**Case presentation:**

A 74-year-old woman presented with a rapidly growing long-standing tumor of the scalp. The tumor was surgically resected. Histologically, the tumor was 25 mm in diameter, and was composed of carcinoma showing a clear pilomatrical differentiation and sarcoma with pleomorphic *spindle cells* and giant cells. Both epithelial and mesenchymal components shared focal cytoplasmic and/or nuclear accumulation of β-catenin based on immunohistochemical analysis, although a mutation of exon 3 of the *CTNNB1* gene was not detected. Fluorescence in situ hybridization analysis revealed gains of chromosomes 9p21, 3, and 7 in both the epithelial and sarcomatous components.

**Conclusions:**

The current case demonstrated characteristic findings of pilomatricoma and further evidence of partial clonality between the carcinomatous and sarcomatous component, suggesting the possibility of malignant transformation of pilomatricoma. Rapid growth of a pilomatrical tumor should warrant the development of a malignant tumor, including CS.

## Background

Carcinosarcoma (CS) is a rare malignant tumor that expresses both mesenchymal and epithelial components. The diagnostic terms of this condition vary, and include sarcomatoid carcinoma, metaplastic carcinoma, and spindle cell carcinoma [1]. Over 100 cases of CS of the skin have been reported [2]; however, only several cases of CS of pilomatrical origin have been reported [2–7]. β-catenin, a downstream effector in the canonical pathway of Wnt, is suggested to be associated with pilomatrical neoplasms, presenting with nuclear and/or cytoplasmic staining within the tumor upon immunohistochemical analysis [8–12]. Mutation of exon 3 of *CTNNB1*, which encodes β-catenin, has also been identified in some pilomatrical neoplasms [8–14]. In addition, LEF1–1, further downstream effector of aberrant β-catenin in Wnt pathway, has been recently tested as a diagnostic marker for pilomatrical tumors (sensitivity = 100%, specificity = 56%) [15]. According to a small number of studies, epithelial and sarcomatous components often share partially identical chromosomal abnormalities, despite their distinct morphologies [3, 16]. Herein, we present a case of pilomatrical CS of an elderly patient with detailed immunohistochemical and chromosomal abnormality profiling.

## Case presentation

**Fig. 1:**
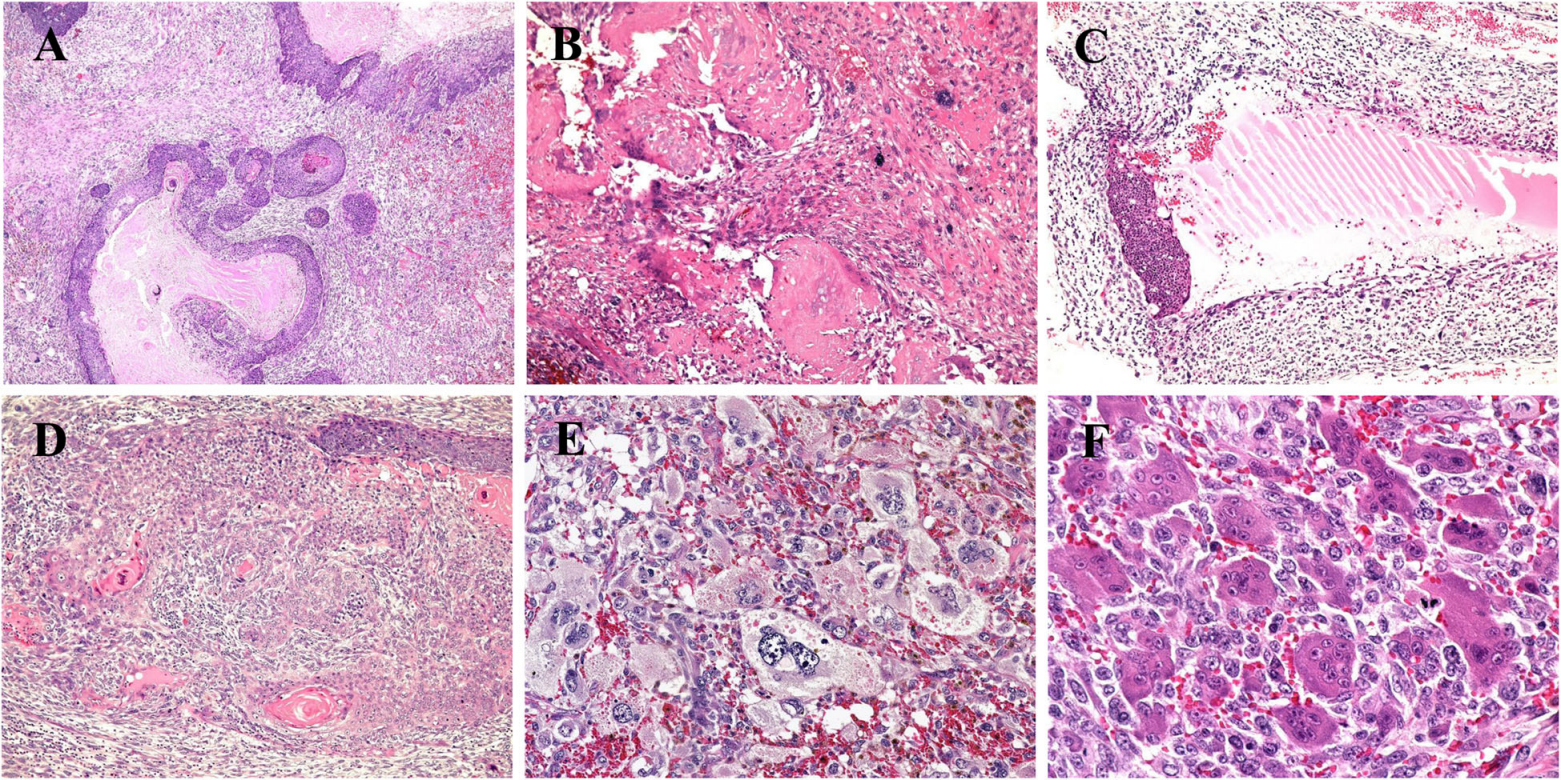
Tumor patterns, hematoxylin eosin staining. HE staining showing **a**: follicular differentiation of carcinoma components (×4), **b**: “shadow cell” and foreign body giant cells (×10), **c**: cystic change with partial coverage in basaloid cell carcinoma × 10, **d**: squamous cell carcinoma component (× 10), **e**: pleomorphic spindle cell sarcoma (UPS) × 10, and **f**: spindle cells with osteoclast-like giant cells (×20)

A 74-year-old female patient presented to the hospital with a tumor on the posterior scalp that had grown rapidly within two months after a long-standing period over the past 10 years. Ultrasonography revealed a well-circumscribed solid mass with a diameter of 25 mm that was partly cystic with calcification. An excision biopsy was performed. Microscopic examination of the lesion revealed a well-demarcated but non-encapsulated dermal mass that had no contact with the superficial epidermis. The tumor showed two closely intermingled components composed of epithelial and mesenchymal malignant tissue (Fig. 1a–f). The epithelial components included basaloid tissue showing follicular differentiation where peripheral atypical basaloid cells transitioned to clear cells and showed degenerative substances at the center of the tumor (Fig. 1a). One area containing eosinophilic “shadow” cells surrounded by foreign body giant cells were observed with some foci of calcification, which was compatible with a remaining benign pilomatricoma (Fig. 1b). A cystic structure was present, which was focally covered with basaloid cells (Fig. 1c). The epithelial component was also admixed with areas of keratinizing atypical squamous cells, indicating squamous cell carcinoma (Fig. 1d). In contrast, the macrophage-rich mesenchymal components included pleomorphic spindle tumor cells as well as multinucleated pleomorphic giant cells (Fig. 1e), which was consistent with undifferentiated pleomorphic sarcoma. Part of the sarcomatous lesion also showed features of giant cell tumor showing elongated mononuclear cells and osteoclast-like multinucleated giant cells which resembled a giant cell tumor of the bone (Fig. 1f). The average mitotic indexes were 14 in one high-power field in the carcinomatous area, and 12 in 10 high-power fields in the sarcomatous areas. Atypical mitotic figures were observed frequently in the sarcomatous area. Vascular or perineural invasion was not observed. The restriction margin was negative.

**Fig. 2:**
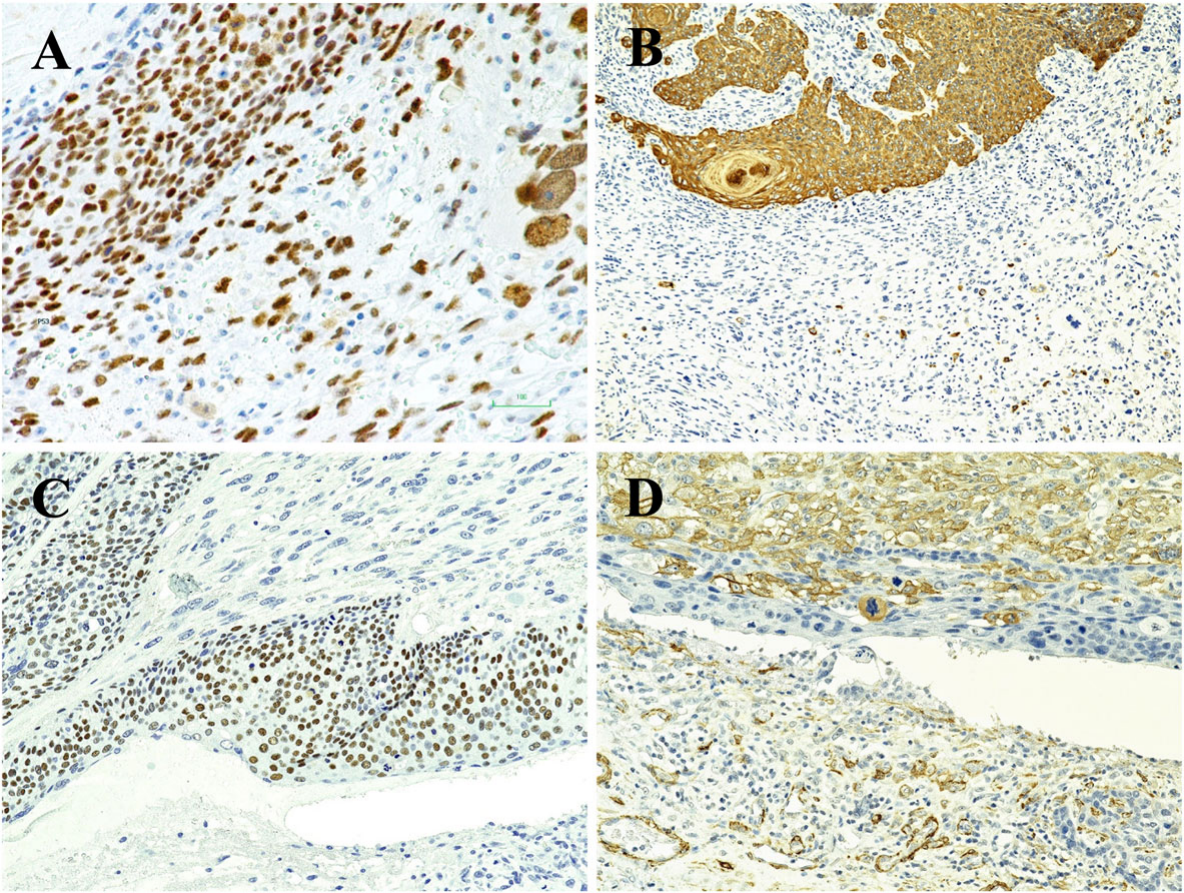
Immunohistochemistry profile. Immunohistochemistry demonstrated **a**: positive P53 staining in the nucleus of both carcinoma and sarcoma components (× 20), **b**: positive AE1/AE3 in the carcinomatous component and scattered-positive in the sarcomatous component (× 4), **c**: positive P63 nuclear staining in the carcinomatous component (× 20), and **d**: positive SMA staining in the pleomorphic spindle cells and negative staining in the carcinomatous component (× 20)

The results of immunohistochemistry are shown in Fig. 2 and Table 1. Both epithelial and mesenchymal components tested positive for P53 (Fig. 2a). The epithelial component tested positive for AE1/AE3 (Fig. 2b), CK5/6, and P40 (data not shown). The nuclear expression of P63 was intense in basaloid cells and showed partial staining in squamous cells (Fig. 2c). The mesenchymal tumor stained diffusely positive for vimentin (data not shown) and partially positive for SMA (Fig. 2d). P63 and AE1/AE3 also stained sparsely positive in the spindle cell element. Macrophages and osteoclast-like giant cells, but not tumor cells stained positively for KP1. A few tumor cells in both epithelial and mesenchymal components stained positively for S100. Based on the above findings, the epithelial component was consistent with pilomatrical carcinoma and the mesenchymal component had features consistent with undifferentiated pleomorphic sarcoma. Consequently, the tumor was diagnosed as one of a pilomatrical CS. The patient has been well without recurrence or metastasis for 45 months.

**Table 1:** Results of immunohistochemical analysis of the tumor

Markers	Tumor component		
	Basaloid epithelial component	Squamoid epithelial component	Sarcoma
AE1/AE3	+	+	+/− scattered
E-cadherin	–	+/− focal	–
CK5/6	+	+	–
P40	+	+	–
P53	+	+	+
P16	+	+	+
P63	+	+/− focal	+/− scattered
CD34	–	–	–
Vimentin	–	–	+
SMA	–	–	+/− focal
Caldesmon	–	–	–
β-catenin	normal type	abnormal type	abnormal type
LEF-1	–	+/− scattered and focal	+

*Abbreviation*: + positive, – negative, *normal type* membranous positive, *abnormal type *cytoplasmic and/or nuclear positive

**Fig. 3:**
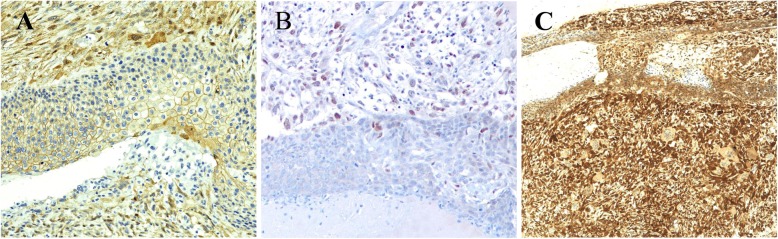
Assessment of β-catenin, LEF-1 and P16. **a**: β-catenin showed normal type (membranous positive) in the carcinomatous component and abnormal type (cytoplasmic and/or nuclear positive) in the sarcomatous component (× 20). **b**: LEF-1 expression is obviously found in nuclei of sarcomatous components but very few in carcinomatous components (× 10). **c**: P16 yielded positive results in the cytoplasm and nuclei of both components (× 10)

**Fig. 4:**
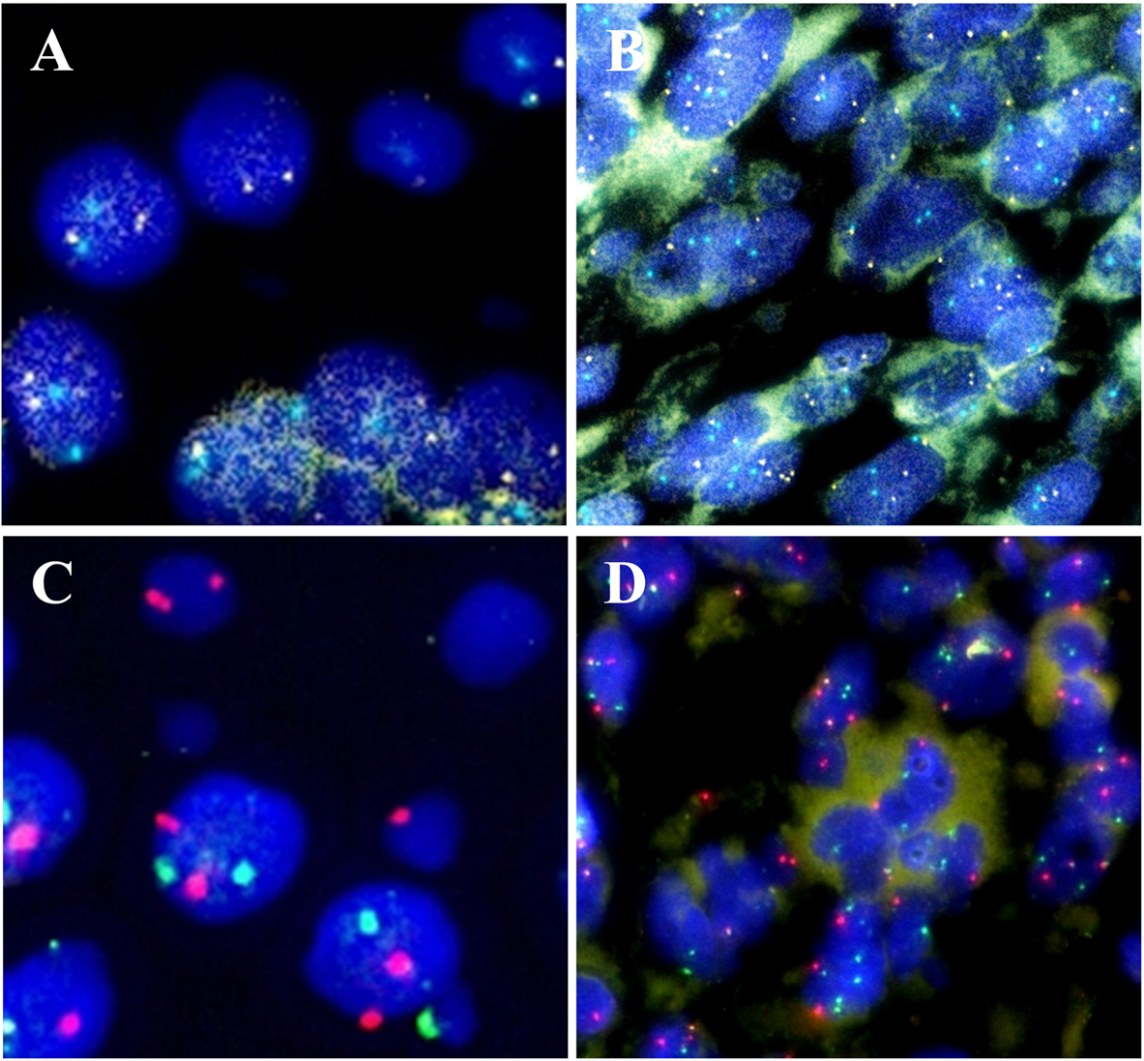
FISH analysis with probes of chromosome 9p21 (gold), 17 (aqua), 3 (red) and 7 (green). **a**, **c**: Benign squamous cell epithelium, where one or two signals per one nucleus is observed for each probe. **b**, **d**: Sarcomatous component of the tumor, where varying copy number gains is observed with an increase in the copy numbers of chromosomes 9p21 (*CDKN2A*, *p16* gene), 3, 7, and 17

For further analysis, we investigated β-catenin and LEF-1 expressions via immunohistochemistry and exons 3, 4, and 5 of the *CTNNB1* gene via polymerase chain reaction assay and sequencing. Cytosolic and/or nuclear staining of β-catenin, termed ‘abnormal type β-catenin expression’ in this study, was partially observed in both components, apart from the sarcomatous tumor component which showed a giant cell tumor pattern (Fig. 3a). LEF1 expression was oberved in nuclei of sarcomatous components but very few in carcinomatous components, which may be transitional cells between basaloid to squamoid (Fig. 3b). A *CTNNB1* mutation was not detected in exons 3, 4, or 5 of the *CTNNB1* gene. Both carcinomatous and sarcomatous components stained diffusely positive for P16 (Fig. 3c). Fluorescence in situ hybridization (FISH) analysis (Fig. 4, Table 2) revealed an increase in the copy number of chromosomes 9p21 (*CDKN2A*, *p16* gene), 3, and 7 in both components. A gain in the copy number of chromosome 17 was present in the sarcomatous lesion, whereas the change was minimal in the carcinomatous area, showing a pattern similar to the normal epithelium surrounding the tumor.

**Table 2:** Comparison of chromosomal aberrations among benign, carcinomatous, and sarcomatous components

Pattern	Benign epithelium (*n* = 6)			Carcinoma (*n* = 13)			Sarcoma (*n* = 21)		
Chromosomes	Mean ± SD	Minimum	Maximum	Mean ± SD	Minimum	Maximum	Mean ± SD	Minimum	Maximum
9p21	0.9 ± 0.14	0.7	1	1.6 ± 0.3	1.1	2.1	1.8 ± 0.5	1.1	2.9
3	1.1 ± 02	0.9	1.3	2.2 ± 0.3	1.8	2.8	2.6 ± 0.6	1.5	4.4
7	1.1 ± 0.2	0.8	1.3	2.3 ± 0.4	1.6	2.9	2.7 ± 0.5	1.8	3.6
17	1.1 ± 0.1	0.9	1.2	1.2 ± 0.2	1.0	1.6	1.6 ± 0.4	1.2	2.6

All values were calculated using the ratio of the centromere or gene on the nuclei

*Abbreviation*: *SD* standard deviation

## Discussion

Primary pilomatrical CS is a rare condition, with only several cases reported in the English literature to our knowledge [2–7]. Summaries of information regarding the present case and other cases of pilomatrical or cutaneous CS are shown in Tables 3 and 4, respectively. Most cases of cutaneous CS show aggressive clinical features with a rapid growth phase varying from days to months, which follows a long-standing phase lasting up to several decades [1, 2, 17, 18]. In our case, the tumor had undergone a 10-year-long slow growth phase followed by rapid growth within 2 months, suggesting that this tumor had clinically transformed from a benign state. Although no exon 3 mutation of *CTNNB1* gene was found, an abnormal expression of β-catenin was focally observed in both the epithelial and sarcomatous components of the lesion. Basaloid pattern showed membranous or cytoplasmic positive while squamous and sarcomatous patterns showed cytoplasmic and nuclear expression. A majority of pilomatrical tumors have been reported to show cytoplasmic and/or nuclear β-catenin expression via immunohistochemistry [8–12]. *CTNNB1* gene mutation, which is considered to force β-catenin expression, has been indicated in the development of follicular tumors; a mutation at exon 3 of the *CTNNB1* gene was also found in 61% [8–10] to 100% [12]. On the other hand, in our case, LEF-1 expression was obviously found in nuclei of sarcomatous components but very few in carcinomatous components. This staining pattern corresponds to the pattern of aberrant beta-catenin expression, suggesting involvement of activated Wnt pathway during sarcomatous transformation. The LEF-1 immunoreactivity in present case is different from the results of Tumminello et al. (2018) which found that LEF-1 was strong and diffuse positive in basaloid cells of pilomatrical tumor [15]. By immunofluorescence analysis, the study of Chan et al. (1999) also showed that LEF-1 expression was prominently found in nuclei of the proliferating cells of pilomatricomas [9]. Although the β-catenin and LEF-1 expression in our case are different from previous reports of pilomatrical tumors, aberrant β-catenin expression in sarcomatous component was concordant with previous reports of pilomatrical CS [5, 6]. Furthermore, previous report of pilomatrical CS investigating both β-catenin expression and *CTNNB1* gene exon 3 mutation showed aberrant β-catenin expression in the tumor without mutation [5], which is consistent with our case. We also note that no mutations were found in exons 4 and 5. These results suggest the presence of alteration other than *CTNNB1* exons 3–5 mutation or other mechanism which activates Wnt pathway during carcinogengesis of pilomatrical CS. Further study is required to clarify the molecular mechanism with accumulation of cases. The results regarding β-catenin expression in previous and our cases are presented in Table 3.

**Table 3:** Summary information of 7 cases of pilomatrical carcinosarcoma

	Hanly et al., 1994 [7]	Scholl et al., 2010 [4]	Clark et al., 2017 [2]	Suyama et al., 2017 [5]	Leecy et al., 2018 [3]	Mori et al., 2019 [6]	Present case
Age	36	23	77	73	87	100	74
Sex	Male	Male	Male	Male	Female	Female	Female
Quiescent period			> 24 months	6 months	4 years	Several decades	10 years
Ulcer	+	–		–	–	–	–
Size (cm)	4		5	2	1.6	1.5	2.5
Site	Cheek	Periauricular	Cheek	Cheek	Hand	Temple	Scalp
Epithelial component	Benign pilomatrixoma	Benign pilomatrixoma	Pilomatrical ^a^ component +BCC	Malignant pilomatricoma	Malignant pilomatricoma	Malignant pilomatricoma	Malignant pilomatricoma + SCC
Mesenchymal component	Undifferentiated pleomorphic sarcoma	Spindle cell sarcoma	Undifferentiated pleomorphic sarcoma	Spindle cell sarcoma	Undifferentiated pleomorphic sarcoma	Spindle cell sarcoma	Undifferentiated pleomorphic sarcoma
β-catenin IHC				Abnormal type in basaloid cells in the epithelial component	Normal type in basaloid cells in the epithelial component	Abnormal type in both component	Abnormal type In both component
CTNNB1 sequencing				Exon 3 wild type			Exon 3, 4, 5 wild type
*Chromosomal* abnormality	–	–	–	–	CGH: Homozygous deletion of chr 17q25, 9p21; 9p24.1-p13.2 and 20p13-p11.1 gain in both components	–	FISH: varying gains of copy number of 9p21, Chr 3, 7, 17 in both component

*Abbreviations*: (+) present, (−) absent, *BCC* basal cell carcinoma, *SCC* squamous cell carcinoma, *Chr* chromosome, *CGH* comparative genomic hybridization, *FISH* fluorescence in situ hybridization

^a^Not specified whether benign or malignant

Blank spaces indicate that no information was available

**Table 4:** Comparison of the present case and other reported cases of cutaneous carcinosarcoma

Authors	Tran et al (2005)		Clark et al (2017)		Present case
Features	Epidermal CS	Adnexal CS	Adnexal CS	Epidermal CS	Pilomatrical CS
Age (mean ± SD)	73 ± 11 (44–91)	58 ± 16 (36–87)	63.3 ± 16.1 (36–92)	76 ± 12 (41–98)	74
Sex ratio (male: female)	17:7	8:10	0.84	2.2	Female
Site at head and neck, prevalence	46%	44%	45.8%	58.6%	Scalp
Ulceration prevalence	48%	33%			Not evident
Epithelial component	BCC 54% SCC 58%	Spiradenocarcinoma 56% Adenocarcinoma 17% Matrical carcinoma 6%	Spiradenocarcinoma 50% Trichogenic carcinoma 25% Pilomatrical carcinoma 1%	BCC 63.2% SCC4 5.9%	Pilomatrical carcinoma+SCC
Mesenchymal component					
UPS (AFX)	70%	56%	74.9%	67.7%	UPS
Osteosarcoma	35%	38%	16.7%	26.4%	

*Abbreviations*: *SD* standard deviation, *CS* carcinosarcoma, *AFX* atypical fibroxanthoma, *UPS* undifferentiated pleomorphic sarcoma, *BCC* basal cell carcinoma, *SCC* squamous cell carcinoma

Blank spaces indicate that no information was available

The association of p16 overexpression and deletion of the *p16* gene (*CDKN2a*, 9p21) with high grade histology and unfavorable prognosis have been recently reported in various cancers [19–24]. In the present case, immunohistochemical analysis demonstrated overexpression of p16 in both components of the tumor (Fig. 3b) and fluorescence in situ hybridization analysis demonstrated an increased copy number of 9p21 (*CDKN2A*, *p16* gene) in both epithelial and sarcomatous components (Fig. 4, Table 2). Furthermore, we observed gains in chromosomes 3 and 7 in both components. Very limited numbers of studies have presented chromosomal aberrations in cutaneous CS, which mainly describes loss of 9p21 or 9p in both carcinomatous and sarcomatous components [3, 16]. Although *the chromosome gained or lost varies among the cases*, they seem to harbor, at least in part, similar chromosomal abnormalities.

There is controversy regarding how CS develops [25–27]. The *collision tumor theory* suggests that two independent tumors collide, while the *composition theory* suggests that the mesenchymal component results from pseudo-sarcomatous reaction within the epithelial malignancy. The *combination theory* posits that both malignant components arise from a common pluripotential stem cell that subsequently undergoes divergent differentiation early in the evolution of the tumor. The *conversion theory* posits that the sarcomatous component results from the transformation of the epithelial component. Our case is suggestive of the conversion theory since the patterns of chromosomal abnormality indicate shared features between the sarcomatous and carcinomatous components of CS, as well as some distinct patterns. In addition, we observed positivity of AE1/AE3 and P63, not only in the epithelial component but also partially in the sarcomatous component. Further, as seen in our case as well as previous reports, CS developed from a seemingly benign lesion after a long-standing period. Taken together, these pieces of evidence suggest the presence of a benign pilomatricoma, which progressed to a carcinoma, and finally transformed into a sarcoma. The presence of a distinct pilomatrical component in our case further supports the conversion theory. However, combination theory cannot be excluded since molecular changes that are common between epithelial and mesenchymal component could have occurred in a primitive tumor cell, which subsequently diverged to different tumor components after additional genetic alterations.

In summary, we have presented a rare case of pilomatrical CS of the scalp. Our morphological and molecular analysis indicated a malignant transformation of carcinoma from a pilomatricoma, which ultimately developed into a sarcoma. As CS presents with aggressive features including remote metastasis which can be fatal, sudden growth of pilomatrical tumors should be managed promptly with caution.

## Data Availability

Not applicable.

## Abbreviations

AFX: Atypical fibroxanthoma (dermal type of UPS)
CS: Carcinosarcoma
UPS: Undifferentiated pleomorphic sarcoma
